# Crawling-induced floor dust resuspension affects the microbiota of the infant breathing zone

**DOI:** 10.1186/s40168-018-0405-8

**Published:** 2018-02-02

**Authors:** Heidi K. Hyytiäinen, Balamuralikrishna Jayaprakash, Pirkka V. Kirjavainen, Sampo E. Saari, Rauno Holopainen, Jorma Keskinen, Kaarle Hämeri, Anne Hyvärinen, Brandon E. Boor, Martin Täubel

**Affiliations:** 1 Environmental Health Unit, National Institute for Health and Welfare, Kuopio, Finland; 2 Department of Clinical Nutrition, Institute of Public Health and Clinical Nutrition, University of Eastern Finland, Kuopio, Finland; 3 Aerosol Physics Unit, Faculty of Natural Sciences, Tampere University of Technology, Tampere, Finland; 4 VTT Technical Research Centre of Finland, Tampere, Finland; 5 Finnish Institute of Occupational Health, Helsinki, Finland; 6 Oulu University of Applied Sciences, Oulu, Finland; 7 Division of Atmospheric Sciences, Department of Physics, University of Helsinki, Helsinki, Finland; 8 Lyles School of Civil Engineering, Purdue University, West Lafayette, IN USA; 9 Ray W. Herrick Laboratories, Center for High Performance Buildings, Purdue University, West Lafayette, IN USA

**Keywords:** Infant exposure, Indoor microbial exposure, Particle resuspension, qPCR, 16S rRNA gene sequencing

## Abstract

**Background:**

Floor dust is commonly used for microbial determinations in epidemiological studies to estimate early-life indoor microbial exposures. Resuspension of floor dust and its impact on infant microbial exposure is, however, little explored. The aim of our study was to investigate how floor dust resuspension induced by an infant’s crawling motion and an adult walking affects infant inhalation exposure to microbes.

**Results:**

We conducted controlled chamber experiments with a simplified mechanical crawling infant robot and an adult volunteer walking over carpeted flooring. We applied bacterial 16S rRNA gene sequencing and quantitative PCR to monitor the infant breathing zone microbial content and compared that to the adult breathing zone and the carpet dust as the source. During crawling, fungal and bacterial levels were, on average, 8- to 21-fold higher in the infant breathing zone compared to measurements from the adult breathing zone. During walking experiments, the increase in microbial levels in the infant breathing zone was far less pronounced. The correlation in rank orders of microbial levels in the carpet dust and the corresponding infant breathing zone sample varied between different microbial groups but was mostly moderate. The relative abundance of bacterial taxa was characteristically distinct in carpet dust and infant and adult breathing zones during the infant crawling experiments. Bacterial diversity in carpet dust and the infant breathing zone did not correlate significantly.

**Conclusions:**

The microbiota in the infant breathing zone differ in absolute quantitative and compositional terms from that of the adult breathing zone and of floor dust. Crawling induces resuspension of floor dust from carpeted flooring, creating a concentrated and localized cloud of microbial content around the infant. Thus, the microbial exposure of infants following dust resuspension is difficult to predict based on common house dust or bulk air measurements. Improved approaches for the assessment of infant microbial exposure, such as sampling at the infant breathing zone level, are needed.

**Electronic supplementary material:**

The online version of this article (10.1186/s40168-018-0405-8) contains supplementary material, which is available to authorized users.

## Background

Early-life microbial exposures support the homeostatic immunological development [1] and have been shown to contribute to the risk of asthma and other immunomodulatory diseases with rising prevalence [2]. In westernized societies, children spend approximately 90% of their time indoors, which emphasizes the health relevance of exposures through indoor air. The immune system goes through rapid changes during early life, adapting and maturing in response to environmental exposures to microbes and allergens it encounters [3]. These patterns of the immune function can persist until adult age and are linked to the etiology of allergy [4]. The connection of early-life microbial exposure to several other diseases, such as childhood asthma, has been studied extensively during the past decades. Farm-related microbial exposure has been shown to have protective effects on childhood asthma [5]. On the other hand, epidemiological and experimental studies have established a link between microbial exposure related to moisture damage and exacerbation of childhood asthma [6–8]. In addition, studies on gut and airway microbiota have indicated that microbial dysbiosis and prevalence and severity of asthma may be connected [9, 10].

A crucial step towards better understanding of the potential role of indoor air microbes is the ability to accurately assess an infant’s exposure, and the factors contributing to it during this critical time window of immunological development. Next generation sequencing approaches offer new possibilities in exploring the possible links between microbial exposures and human microbiota in health and disease. Epidemiological studies exploring early-life exposure to microbes and their health effects typically use settled dust samples collected from flooring, mattresses, and other surfaces to assess the microbial content and diversity that small children are being exposed to. These sampling approaches are convenient and allow application in large epidemiological studies, as they do not require complex sampling equipment and home visits to collect the samples, and the sampling can be carried out by the study participants themselves. Floor and mattress dust samples represent long-term exposure more comprehensively than short-term air samples, which are affected by momentary variation generated by human activities and outdoor sources, among others [11, 12]. However, these methods have certain limitations in estimating the microbial composition and levels in the infant breathing zone (IBZ). Floor dust is only moderately representative of the microbial content in indoor air [13, 14]. Passive sampling of dust settling on elevated surfaces above floor level has therefore been introduced for indoor microbial exposure assessment, with microbial determinations based on such samples generally better reflecting the microbial content of indoor air [15], although the use of settled dust samples to represent indoor air exposure is disputed [16, 17]. All of these commonly used methods have deficiencies in estimating the real-life situation of infant inhalation exposure to microbes, which is affected also by the transient activities of children and the resuspension of microbes that their movements induce. Only few studies have tried to fill this gap, with determinations of particulate matter, fungal spores, certain allergens, and endotoxins included in those assessments [18–20].

Differences in children’s activities compared to adults have a crucial effect on the quantity and quality of their exposure to environmental agents [21]. Infants and toddlers spend their time in different microenvironments, often in very close proximity to flooring and bedding surfaces, for example, when they crawl and play. Children below the age of five also ingest soil, house, and street dust (typically 100 mg/day) [22]. Their movements stir up and resuspend floor dust, which is likely to contribute significantly to an infant’s inhalation exposure to coarse-mode particles (> 1 μm in aerodynamic diameter), microbes, pollen, and allergens [23].

The microbial samples commonly used in studies to investigate the health effects of indoor microbes, such as settled dust on indoor surfaces, may mischaracterize the inhaled microbial exposures in small children. In this study, we seek to gather hitherto non-existing knowledge on the characteristics of the infant breathing zone microbiota raised to the air from floor by crawling or walking movements. We performed chamber experiments with a simplified mechanical baby robot crawling over carpeted flooring and compared infant breathing zone samples with the commonly used proxies for microbial exposure—bulk air, representing the adult breathing zone and floor dust samples.

## Methods

### Study design and controlled chamber experiments

The study was designed with the aim to evaluate human inhalation exposure to resuspended floor dust particles of biological origin in controlled chamber measurements. The study was focused on quantitative and qualitative analyses of the microbiota in the IBZ during crawling resuspension sequences of a custom-built 4-kg simplified mechanical crawling infant. In parallel, we carried out determinations of dust collected from the carpets on which the robotic infant performed its crawling sequences on and—in a subset of experiments—the bulk air in the chamber, representing the adult breathing zone (ABZ). We also compared resuspension scenarios of the infant crawling and an adult walking over the carpets. The movements of the robot infant mimicked a belly crawl. A video of the robotic baby crawling can be found at https://figshare.com/articles/Crawling_Infant_Resuspension_Study/5307337 (10.6084/m9.figshare.5307337).

Measurements were conducted in an 81.4-m^3^ chamber operated at a ventilation rate of 0.66 h^− 1^ and supplied with HEPA-filtered air. The chamber was maintained at a temperature of 23.11 ± 0.77 °C and a relative humidity of 23.89 ± 4.77% during the measurements. The crawling and walking sequences were conducted on carpets borrowed from residents in the Helsinki-Espoo area of Finland. Carpets were used as is, without the use of artificial test dust, to allow for a more realistic exposure scenario. The residents were asked to refrain from vacuuming their carpets for a minimum period of 2 weeks, prior for their use in the chamber experiments.

**Fig. 1:**
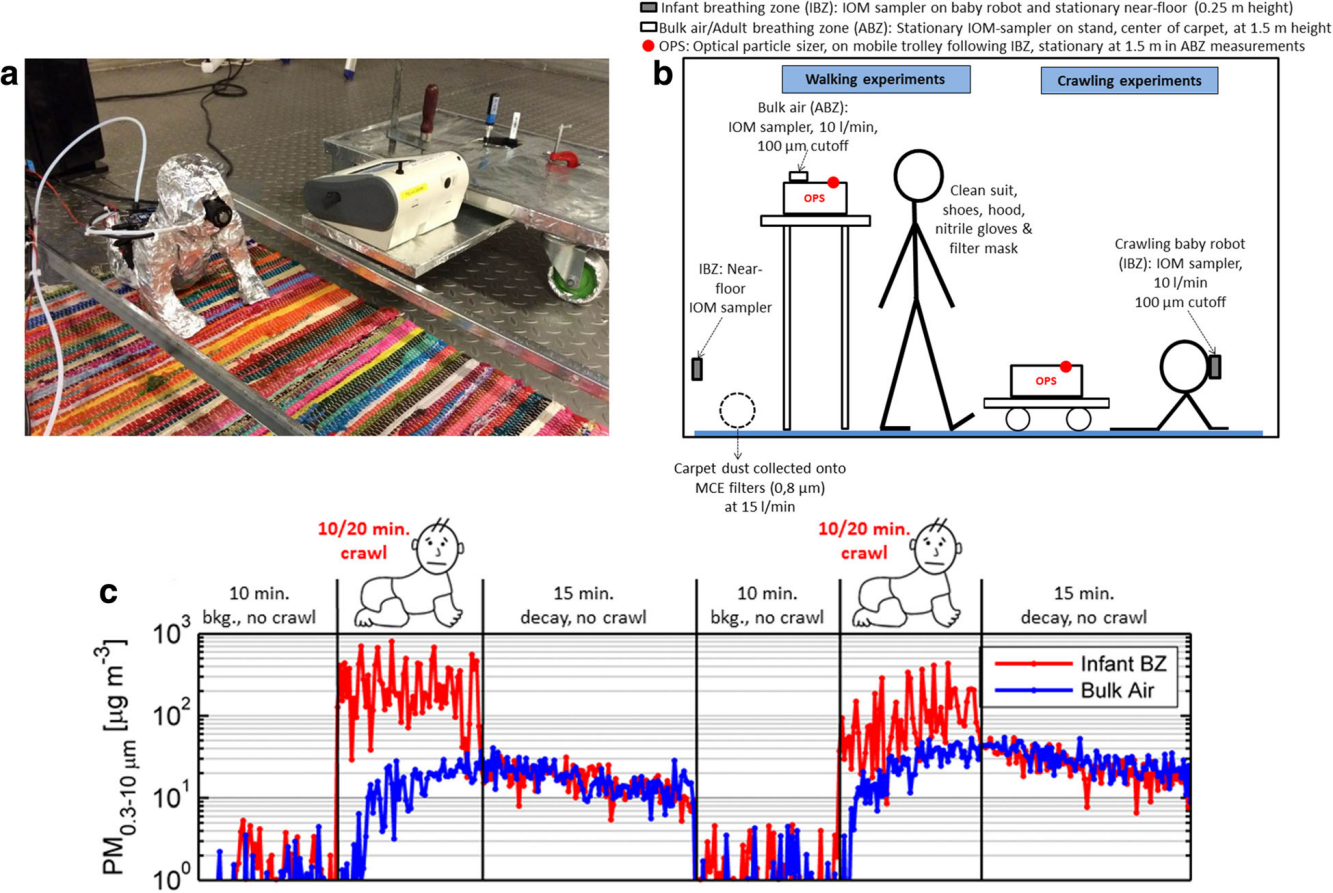
The simplified mechanical crawling infant robot with IOM sampler mounted to the robot’s head and optical particle sizer (OPS) on mobile trolley (**a**), experimental setup of the chamber experiments (**b**), and example sequence of particle mass concentrations monitored with an OPS in the infant breathing zone and in the bulk air during two crawling events (**c**)

In total, 17 such carpets were used in individual experiments. Dust from each carpet was collected onto one 37-mm filter cassettes with 0.8-μm pore-sized MCE (mixed cellulose ester) filters after the crawling or walking events by vacuuming an area of 25 cm × 25 cm of the carpet with a pump at a flow rate of 15 L/min. Active air samples were collected onto 25-mm 0.8-μm pore-sized MCE filters using an IOM (Institute of Medicine) sampler operated at 10 L/min (cutoff ~ 100 μm). The IOM sampler was mounted on the head of the mechanical crawling infant, at a height of approximately 25 cm above floor level (Fig. 1a), and connected to a pump external to the chamber. All filters were conditioned at 23 °C/50% RH for 48–72 h prior to weighing before and after the experiments. Repeated crawling sequences of 10 to 20 min were combined into one integrated IBZ air sample, representing 60–100 min of total crawling action. For five carpets, we collected air samples for microbial determinations also in the bulk air at a height of 1.5 m (ABZ) concurrently with those at the IBZ. For these stationary measurements, IOM samplers and filters used were as specified in Fig. 1b.

The same five carpets were used in five additional experiments with a person walking over the carpets to compare crawling- and walking-induced resuspension scenarios and their effect on IBZ and ABZ microbial concentrations (Fig. 1b). The adult volunteer (height 188 cm, weight 80 kg) wore a full clean suit outfit with booties (DuPont™, Tyvek Pro-Tech Suit Classic), hood, nitrile gloves, and a filter mask to prevent particle shedding from the human envelope. The same carpet dust and IOM active air sampling was performed for the walking experiments, as described for the crawling sequences, with the only difference being that the IBZ was represented by a stationary IOM sampler located at 25 cm above floor level immediately adjacent to the carpet.

The measurements for determining the microbiota in the IBZ and ABZ during resuspension events were conducted in parallel with extensive real-time monitoring of total and fluorescent particle size distributions in both sampling locations, using an optical particle sizer (OPS, model 3330, TSI, Inc., USA) and a laser-induced fluorescence-based instrument, the BioScout™ [24]. Particle size distributions, size-resolved resuspension and emission rates, regional lung deposited dose rate analysis, and additional details of the experimental setup and carpets are presented by Wu et al. [25] and (Fu et al: The Infant Playpen Effect: Crawling-Induced Dust Resuspension as a Major Source of Particulate Matter in the Infant Breathing Zone, in preparation). An example of particle mass concentrations (PM_0.3 − 10 μm_) in both the IBZ and ABZ during the infant crawling sequence is illustrated in Fig. 1c.

### Sample processing and DNA extraction

All collected filter and dust samples were stored and transferred cooled, and processed within 2 weeks after sample collection at the analyzing laboratory at THL. Minor differences in storage times between samples of different experiments are acknowledged. However, corresponding carpet dust, infant breathing zone, and adult breathing zone samples within an experiment, being used for the main comparison in this analysis, have been always stored for the same duration prior sample processing. Carpet dust was removed from the 37-mm filter cassettes and sieved through a sterile strainer (pore size 1 mm × 1 mm) to remove coarse particles and homogenize the dust sample. The dust weight was accurately determined, and 20 mg (± 5 mg) of dust was transferred into glass bead tubes for subsequent DNA extraction. The 25-mm filter membranes from the IOM samplers that were used for active air collection were directly transferred into glass bead tubes for subsequent DNA extraction.

DNA was extracted and cleaned from the samples using a Chemagic DNA Plant Kit (PerkinElmer chemagen Technologie GmbH, Germany) and a KingFisher™ mL DNA extraction robot (Thermo Fisher Scientific, Inc., Finland). The extraction was started with a bead-milling step for mechanical cell disruption [26], using a MiniBeadbeater-16 for one minute (Biospec Products, Inc., USA). Deoxyribonucleic acid sodium salt from salmon testes (Sigma-Aldrich Co., USA) [27] was added to the samples prior to extraction as an internal standard, in order to assess and correct for the presence of inhibitors and the performance of the DNA extraction. DNA was stored at − 20 °C until subsequent analysis.

### PCR and sequencing of the bacterial 16S rRNA gene

The extracted DNA was shipped frozen to the sequencing partner LGC Genomics GmbH (Germany), who performed the library preparation and sequencing. A pre-amplification of sample DNA was performed using primers 341F (CCTACGGGNGGCWGCAG) [28] and 1061R (CRRCACGAGCTGACGAC) [29]. These PCRs included approximately 5 ng of DNA extract, 15 pmol of each primer in 20 μL volume of MyTaq buffer containing 1.5 units MyTaq DNA polymerase (Bioline GmbH, Luckenwalde, Germany), and 2 μL of BioStabII PCR Enhancer (Sigma-Aldrich Co.). Pre-amplification PCRs were carried out for 20 cycles using the following parameters: 2 min 96 °C predenaturation, 96 °C denaturation for 15 s, 50 °C annealing for 30 s, 70 °C extension for 90 s, and hold at 8 °C. The V4 region of the 16S rRNA gene was then amplified using 515F/806R primers for 20 cycles [30]. The PCRs included 1 μL of pre-amplification product, 15 pmol of each forward primer 515F N_1–10_GTGCCAGCMGCCGCGGTAA and reverse primer 806R N_1–10_GGACTACHVGGGTWTCTAAT (N_1–10_ indicates the 10 nucleotide inline barcodes) in 20 μL volume of MyTaq buffer containing 1.5 units MyTaq DNA polymerase (Bioline GmbH, Luckenwalde, Germany), and 2 μL of BioStabII PCR Enhancer (Sigma-Aldrich Co.). For each sample, the forward and reverse primers had the same 10-nt barcode sequence. The following parameters were used for the 20 cycles of PCRs: 2 min 96 °C predenaturation, 96 °C denaturation for 15 s, 50 °C annealing for 30 s, 70 °C extension for 90 s, 70 °C final extension for 90 s, and hold at 8 °C. Approximately 20 ng amplicon DNA of each sample were pooled for up to 48 samples carrying different barcodes. The amplicon pools were purified with one volume Agencourt AMPure XP beads (Beckman Coulter, Inc., IN, USA) to remove primer dimer and other small mispriming products, followed by an additional purification on MinElute® columns (QIAGEN GmbH, Hilden, Germany). Approximately 100 ng of each purified amplicon pool DNA was used to construct Illumina libraries using the Ovation® Rapid DR Multiplex System 1-96 (NuGEN Technologies, Inc., CA, USA). Illumina libraries (Illumina, Inc., CA, USA) were pooled and size selected by preparative gel electrophoresis.

Sequencing was performed on a MiSeq® with V3 chemistry (Illumina) resulting in paired-end reads with a length of 300 bp each. The libraries were demultiplexed using Illumina’s bcl2fastq Conversion Software v1.8.4 (https://support.illumina.com/downloads/bcl2fastq_conversion_software_184.html) and all sequence reads processed with custom Python™ v2.7.6 scripts to sort them by sample removing barcode and amplicon primer sequences. Adapter sequences were removed from the 3′ end of reads with a proprietary script discarding reads shorter than 100 bp.

### Bioinformatics analyses

All 16S rRNA gene targeted amplicon reads were processed and analyzed using QIIME™ (Quantitative Insights Into Microbial Ecology) software version 1.9.1 [31] (see Additional file 1: Table S1 for the mapping file used). The raw reads were initially preprocessed by removal of artificial sequences including adapters by Cutadapt software [32] followed by trimming of bad quality reads and ambiguous sequences by Trimmomatic software [33]. The preprocessed reads were merged using FLASH (Fast Length Adjustment of SHort reads) software [34]. UCHIME [35] was employed to remove chimeras in the preprocessed reads using the USEARCH algorithm [36]. Alignment was done using pynast [37] with greengenes database [38] and sorted with > 97% similarity into operational taxonomic units (OTUs) using open reference OTU picking approach. Taxonomic classification was obtained using the RDP classifier [39]. Alpha- and beta-diversity values were calculated by standard metrics, such as Chao1, Simpson, Shannon, and Unifrac [40], available in QIIME. Negative and positive (bacterial mock) controls were included in the sequence processing of the samples in order to inform the decision on alpha rarefaction—done at 5926 sequences per sample—and to exclude samples closely clustering to negative control samples in PCoA plots. NMDS plots were done using ggplot2 package [41] from *R* programming language version 3.2.4 [42] to display the beta-diversity differences between the samples.

### Quantitative PCR analyses

Quantitative PCR (qPCR) utilizing previously published qPCR assays was used for quantitation of following bacterial and fungal groups: Gram-positive and Gram-negative bacteria [43]; group of *Penicillium* spp., *Aspergillus* spp., and *Paecilomyces variotii,* total fungal DNA and *Cladosporium herbarum* [44]; and internal standard salmon testis DNA [27]. QPCR reactions were performed as written in the original publications with minor modification. In the bacterial duplex assay (Gram-positive and Gram-negative bacteria), 20-μL reaction mix were used, consisting of 10 μL of Environmental Master Mix (Applied Biosystems Inc., Foster City, CA, USA), 1.5 μL bovine serum albumin (2 mg/ml), 1 μL of each forward and reverse primers, 0.4 μL of both TaqMan probes, 3.7 μL of nuclease-free water (HyClone Laboratories Inc., UT, USA), and finally 2 μL of template DNA. Reactions were performed in 0.2 mL 96-well plates (Agilent Technologies Inc., CA, USA) with the Stratagene Mx3005P qPCR System (Agilent Technologies Inc., CA, USA) equipment. Positive (bacterial and fungal mock communities including qPCR target strains) and negative controls (reagent control), as well as no template controls, were included in the qPCR runs. Numbers of microbial cell equivalents (CE) in the samples were calculated using relative quantification as described earlier [45] and normalized for sampled air volume for active air samples (CE/m^3^) and amount of dust (CE/mg) or carpet area (CE/m^2^) for carpet dust samples. In Additional file 1: Table S2, details on primer sequences and qPCR conditions are provided.

### Statistical analyses

Differences in Chao1 estimated species richness and Shannon diversity, as well as differences in relative abundance of individual taxa in carpet dust versus the corresponding IBZ samples, were analyzed using pairwise sample comparison. Non-parametric statistical methods were used because the microbial data was not normally distributed. The differences between carpet dust and IBZ microbiota (Chao1 richness, Shannon index, genus level taxa summaries) were tested using Wilcoxon signed-rank test (for matched pair data) [46], and dependence between these two sample types were examined with Spearman rank-order correlations [47]. Similarly, Spearman rank-order correlations were used to examine dependence between qPCR levels in the different sample types. Statistical analyses were performed using SAS software (version 9.3, SAS Institute Inc., Cary, NC, USA). The correlations figures were plotted in ggplot and corrplot packages in R programming language version 3.2.4.

Differences in bacterial abundance weighted Unifrac distance were calculated using ANOSIM statistical test [48] available in QIIME platform. LEfSe [49] based on linear discriminant analysis was used for the identification of discriminate features (OTUs) between carpet dust and IBZ samples.

## Results

### Microbial concentrations in carpet dust

The dust loads from the 17 carpets used in the chamber experiments varied between 0.72 and 20 g/m^2^ (median 4.0 g/m^2^). Microbial loads (CE/m^2^), i.e., microbial content calculated per carpet area, in floor dust determined with qPCR assays targeting several fungal and bacterial groups differed by at least two orders of magnitude among the carpets (Additional file 2). Loading of Gram-negative bacteria showed the greatest variation among the carpets, varying over three orders of magnitude. When microbial levels in carpet dust were calculated per milligram of sampled dust, differences among the carpets were somewhat smaller but still spanned two orders of magnitude or more (see Additional file 1: Table S3).

### Correlations of microbial concentrations in carpet dust and infant breathing zone

**Fig. 2:**
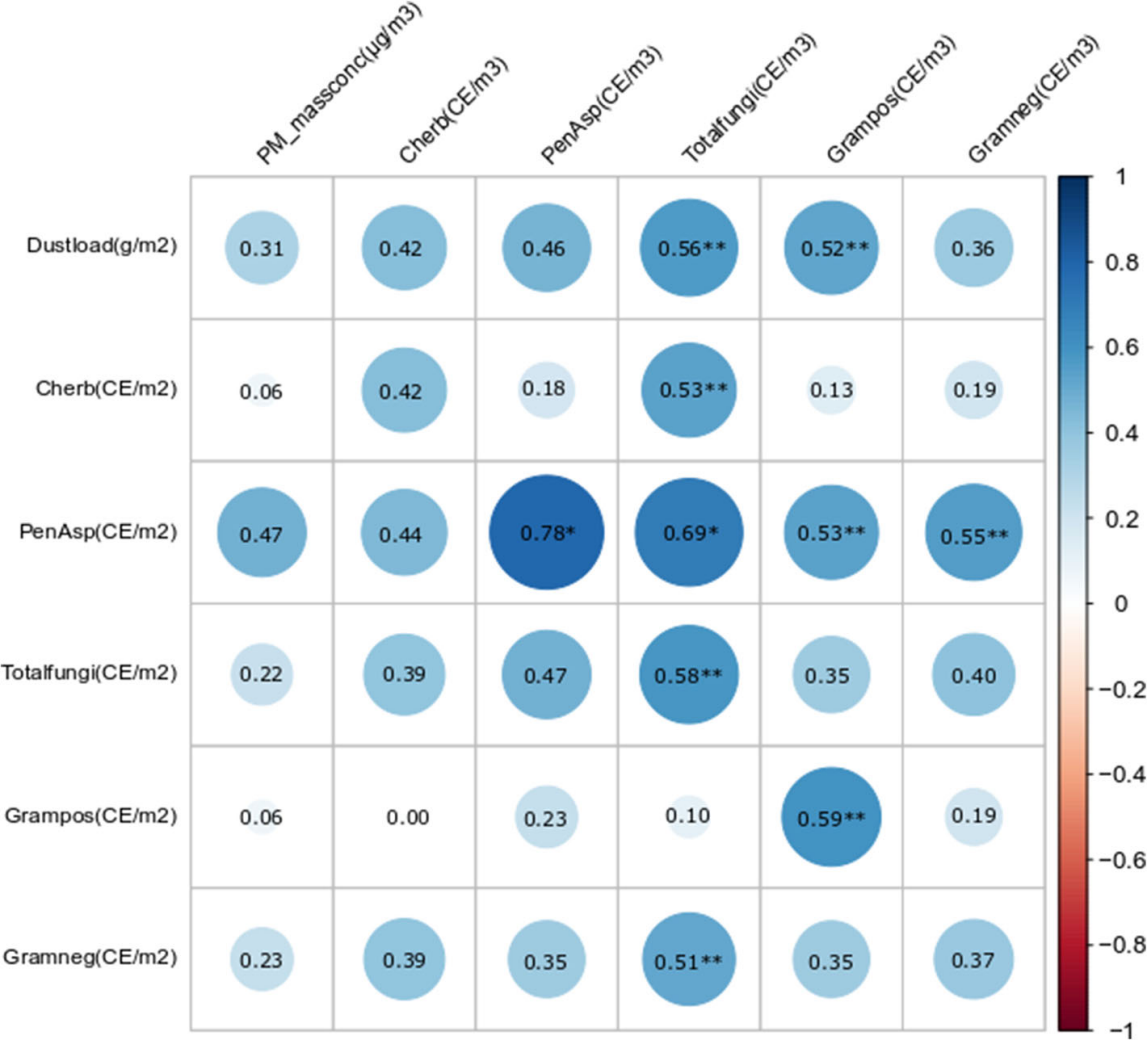
Spearman rank-order correlations (rho) between microbial and dust loads in carpet versus their concentration infant breathing zone during crawling experiments on 17 carpets. Microbial concentrations were determined with qPCR and expressed as load in dust (CE per square meter of carpet) versus air concentrations (CE per cubic meter of air). The size and shading of the circles indicate the strength of the correlation in addition to the correlation coefficients shown (rho). The significant correlations are marked with asterisks. The correlations of dust load (Dustload), PM mass concentration (PM_massconc), *Cladosporium herbarum* (Cherb), Penicillium/Aspergillus group (PenAsp), total fungal DNA (Totalfungi), and Gram-positive and Gram-negative bacteria (Grampos, Gramneg) were explored

Spearman rank-order correlations between microbial loads in carpets and in the IBZ were calculated for different fungal and bacterial groups based on the qPCR analysis (Fig. 2). In general, carpet microbial loads (CE/m^2^) showed higher correlations with the microbial concentrations in the IBZ (CE/m^3^) than when normalizing the dust measurements by the mass of dust, i.e., expressing the data as microbial concentrations (CE/mg of dust) (Additional file 3). The correlations between microbial loads in floor dust and in the IBZ air were significant and strong for *Penicillium*/*Aspergillus* group (0.78), moderate for Gram-positive bacteria and total fungi (0.59), and non-significant and moderate or weak for *C. herbarum* (0.43) and Gram-negative bacteria (0.38). The mass of settled dust in the carpets (dust load, g/m^2^) correlated moderately with levels of most microbial markers in the IBZ (0.42–0.56) and weakly with Gram-negative bacteria and particle mass concentration in the IBZ.

### IBZ versus bulk air (ABZ) levels during resuspension by crawling and walking

Fungal and bacterial concentrations were on average 9- to 21-fold higher in the IBZ (25 cm height) than in the ABZ (1.5 m height) during crawling experiments (Table 1). While the fold difference varied between the different carpets, the concentrations were always higher in the IBZ than ABZ. The fold differences were greater for bacteria compared to fungi. During adult walking experiments, the differences in microbial concentrations between the IBZ and ABZ were distinctly smaller than during the crawling experiments (maximum 2-fold). The IBZ microbial exposure levels to different microbial groups were on average (over 5 experiments) 1.3- to 3.4-fold higher during crawling than during walking sequences (see Additional file 1: Table S4).

**Table 1:** Means, minimum, and maximum of particulate matter (PM_100_ [μg/m^3^]) and microbial levels (cell equivalents/m^3^) in infant breathing zone (IBZ) versus adult breathing zone (ABZ) during both five individual crawling and walking experiments on different carpets and corresponding ratios of IBZ versus ABZ

	PM or microbial group	Infant breathing zone (IBZ)		Adult breathing zone (ABZ)		Ratio of IBZ to ABZ	
		Mean	Min-max	Mean	Min-max	Mean	Min-max
Infant crawling experiments	PM_100_ conc. [μg/m^3^]	210	87–310	73	18–150	4.6	1.3–12
	Cherb [CE/m^3^]	13	4.9–29	1.7	0–8.5	–	–
	PenAsp [CE/m^3^]	250	100–560	34	0–140	7.9	4.1–13
	Total fungi [CE/m^3^]	450	91–1100	92	0–370	9.4	3.0–20
	Gram pos [CE/m^3^]	14,000	1900–23,000	700	400–970	21	4.8–47
	Gram neg [CE/m^3^]	8500	760–17,000	1300	400–3000	13	1.1–43
Adult walking experiments	PM_100_ conc. [μg/m^3^]	50	22–79	43	17–70	1.3	0.4–1.9
	Cherb [CE/m^3^]	3.7	0–9.3	4.5	0–15	–	–
	PenAsp [CE/m^3^]	170	0–510	120	30–334	2.1	1.5–3.9
	Total fungi [CE/m^3^]	310	37–1100	200	30–640	1.2	0.6–1.7
	Gram pos [CE/m^3^]	5200	1400–9400	3600	830–6800	1.4	0.7–2.0
	Gram neg [CE/m^3^]	3400	2200–7100	2300	1100–6100	1.8	1.2–2.2

### Bacterial community composition of carpet dust, IBZ, and ABZ

**Fig. 3:**
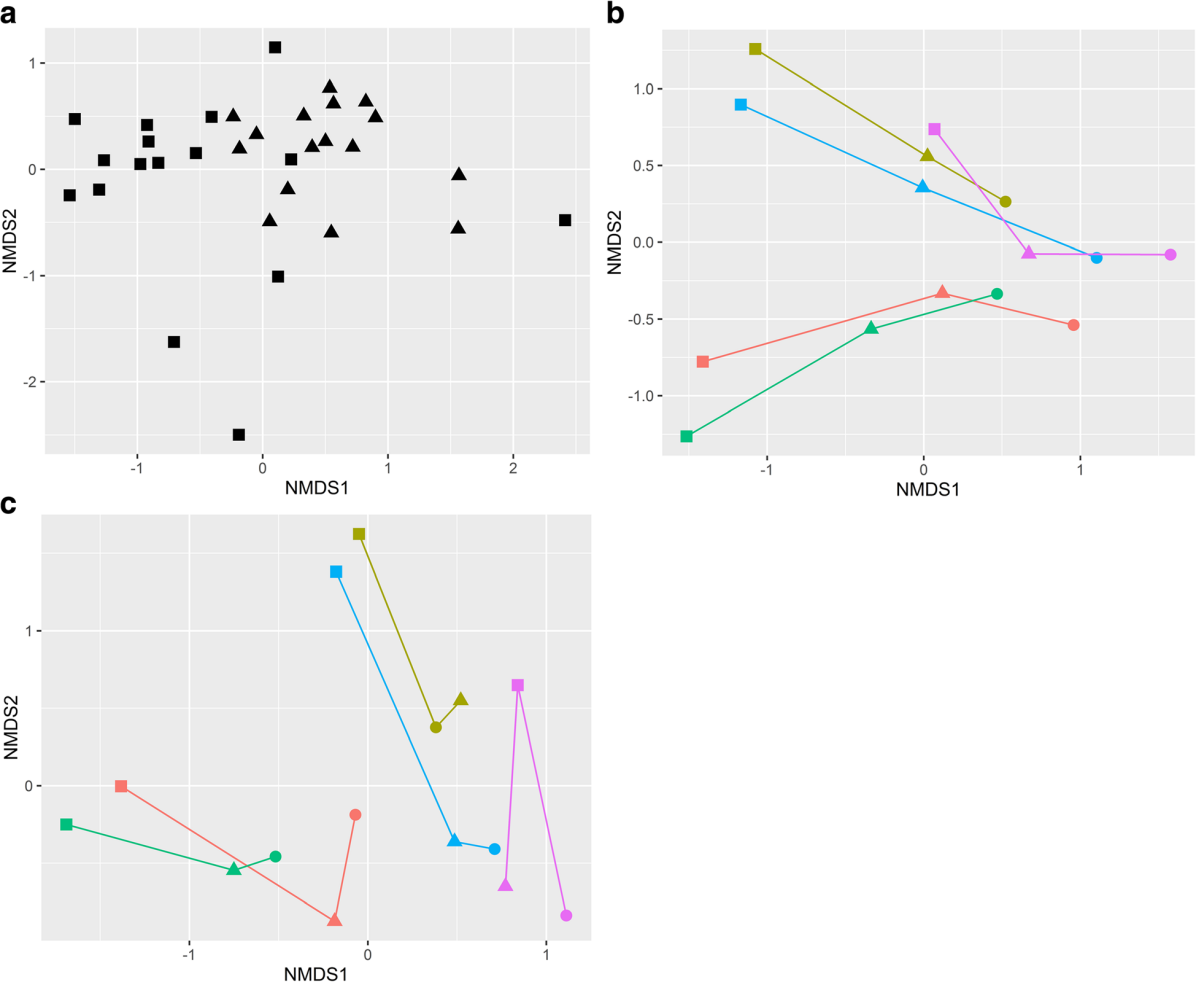
Differences in bacterial community composition using weighted bacterial Unifrac distance, visualized in NMDS plots. Carpet dust (squares) and corresponding air samples (triangles) collected in the infant breathing zone (IBZ) during crawling experiments on 16 different carpets (**a**). Carpet dust (squares), IBZ (triangles), and ABZ (circles) air samples collected in parallel during crawling experiments on five carpets (**b**) and during walking experiments on the same carpets (**c**). The carpet dust and corresponding IBZ and ABZ samples from the individual experiments are connected for clarity in panels **b** and **c**

**Fig. 4:**
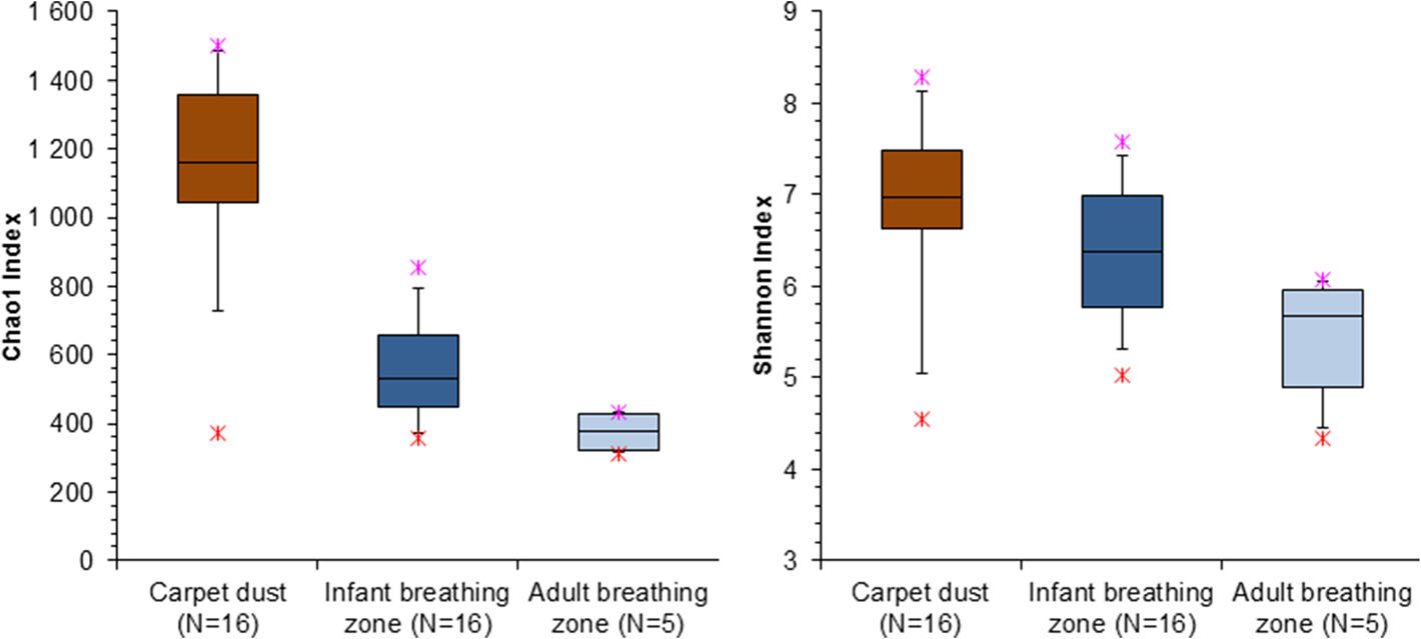
Bacterial Chao1 estimated species richness and Shannon diversity in carpet dust, infant breathing zone, and adult breathing zone (boxes represent the 25th, 50th, and 75th percentiles and whiskers 5th and 95th percentiles; stars mark minimum and maximum values)

**Fig. 5:**
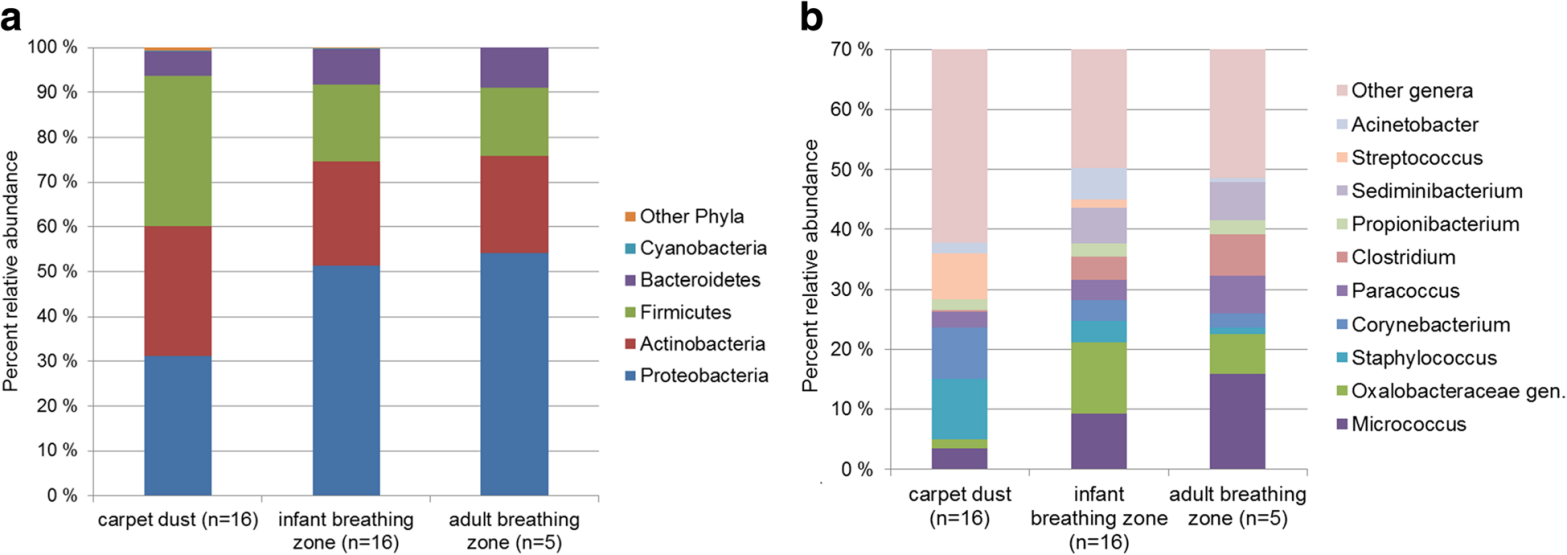
Median relative abundance of the most abundant phyla in carpet dust, IBZ, and ABZ samples (**a**) and median relative abundance of the top 10 (over all sample types) most abundant bacterial taxa on genus level by sample type (**b**). To gain higher resolution on individual genera presented, the *y*-axis of panel **b** has been cut off at 70%

Differences in abundance weighted bacterial β-diversity between carpet dust, IBZ, and ABZ during crawling and walking resuspension experiments are illustrated in Fig. 3. The bacterial composition (based on weighted UniFrac distance) differed significantly (*R* = 0.378, *p* = 0.001 in ANOSIM analysis) between the carpet dust and IBZ samples in the crawling experiments (Fig. 3a). We focused our analyses here on weighted UniFrac distance, but also, in unweighted β-diversity, carpet dust and IBZ microbiota differed significantly and the effect size was even larger than using the weighted distance matrix (*R* = 0.552, *p* = 0.001). We observed significant correlation of NMDS axis score 2 between carpet and IBZ samples (rho = 0.69, *p* = 0.003), indicating a separation of the individual carpet experiments along NMDS2. Floor dust, IBZ, and ABZ samples formed separate clusters along NMDS1 when comparing the bacterial composition of samples in the set of five crawling experiments (Fig. 3b). In addition, the individual carpet experiments separated visibly along NMDS2, but the low sample number prohibited a meaningful correlation analysis. During walking experiments, the microbial community distance of IBZ and ABZ samples was much less pronounced compared to the crawling experiments (Fig. 3c). Bacterial Chao1 estimated richness and Shannon diversity index were highest in the carpet dust, and lowest in the ABZ (Fig. 4), significantly (*p* < 0.001) different for both Chao1 estimate and Shannon index between carpet dust and IBZ (not calculated for IBZ and ABZ due to low sample numbers). Neither richness (rho = 0.24) nor diversity (rho = 0.27) correlated significantly between the carpet dust and the IBZ samples. *Firmicutes*, *Actinobacteria*, and *Proteobacteria* were the dominant phyla in carpet dust. In the vertical gradient above the carpet, from the IBZ to ABZ, *Proteobacteria* was increasingly abundant with distance from the floor level, due to the lower relative abundance of *Firmicutes*, which decreased from the IBZ to the ABZ air (Fig. 5a). At the phylum level, only the relative abundance of *Actinobacteria* correlated significantly between the carpet dust and IBZ sample pairs (Spearman’s rho 0.62, *p* = 0.01).

Differences in the relative abundance of the most abundant (across sample types) bacterial genera were apparent (Fig. 5b): *Staphylococcus*, *Corynebacterium*, and *Streptococcus* were dominant in carpet dust samples, but not so in the IBZ and ABZ. *Micrococcus* and *Oxalobacteraceae gen.* were the genera with highest relative abundance in the IBZ and ABZ (Fig. 5b), and all of these genera were far less abundant in the carpet dust than in the air samples. Spearman rank-order correlation of relative abundance of these genera in carpet dust and IBZ revealed significant (< 0.05), moderate to good correlations (rho = 0.51 to 0.73) for six groups and non-significant, weak, or moderate correlations (0.23–0.48) for the other four genera (Additional file 1: Table S5).

LEfSe analysis was performed to identify the differential features (OTUs) in carpet dust versus the IBZ. In total, 371 OTUs had significantly different relative abundance in IBZ and carpet dust (Additional file 1: Table S6). The majority of taxa with greater relative abundance in carpet dust were allocated to *Firmicutes*, *Actinobacteria*, *Proteobacteria*, and *Bacteroidales* phyla, whereas OTUs enriched in the IBZ were mostly allocated to *Proteobacteria*.

## Discussion

In this study, we investigated the microbiota in the breathing zone air of a robotic infant as it crawled across the floor and monitored the effects of floor dust resuspension on an infant’s inhalation exposure. We observed unique attributes in regard to the compositional and quantitative aspects of microbial exposure in the IBZ that are not predicted well by surrogate measurements that rely on floor dust or bulk air sampling in the ABZ.

The effects of an infant crawling and a person walking on floor dust resuspension and consequently on the microbial composition in the IBZ air were investigated for the first time in our study. Our results from the qPCR and compositional bacterial amplicon sequencing analyses suggest that the IBZ microbial composition during crawling has characteristics distinct from the bulk air and floor dust microbiota. We observe that the predictive value of microbial levels determined from floor dust on levels in the IBZ is limited and depends on the microbial group that is measured. While Gram-negative bacteria did not correlate significantly between carpet dust and the IBZ, Gram-positive bacteria and some of the more general fungal markers did. The microbial levels in IBZ (per m^3^ of air) correlated better with the microbial loads (i.e., microbes per floor area) than the microbial concentrations (i.e., microbes per milligram of dust) determined from carpet dust. This is probably because loads take into account the amount of dust—including the microbes associated with it—that is actually present on the carpet and available for resuspension, even though we only observed weak correlations between carpet dust load and pm PM_100_ mass concentration in the IBZ. In epidemiological studies, microbial measurements are often expressed as concentrations per milligram of dust, being more reflective of the composition of the dust and less of the amount of the potential exposure. Our results support reporting microbial loads rather than concentrations when carpet dust measurements are performed to infer inhalation exposure.

In the compositional analyses based on bacterial 16S rRNA gene amplicon sequencing, we found a clear separation of carpet dust from IBZ samples. The bacterial composition was significantly different for these two sample types, also visible in the relative abundance of the predominant taxa. At the phylum level, we observed a shift from *Firmicutes* dominance in floor dust to a *Proteobacteria* dominance in the IBZ. At the genus level, *Staphylococcus* and *Corynebacterium*, among others, were dominant in floor dust, but less so in the IBZ. Both of these genera are representatives of the human skin microbiota, and we hypothesize that their higher relative abundance in carpet dust may be due to these bacteria being frequently carried on larger skin flakes, or squames (40 × 30 × 2 μm in size) [50]. Skin flakes of this size can be readily collected via common floor dust collection techniques, as well as resuspended during human activity, but may not remain airborne long enough to reach the IBZ or ABZ due to their high settling velocities (> 0.1 cm/s). We also found that Chao1 estimated bacterial richness and Shannon diversity did not correlate between carpet dust and the IBZ. This is an important finding, given that the diversity of microbial exposures early in life has become a central topic in asthma and allergy research. Our results taken together indicate that floor dust is a poor proxy of the microbial content inhaled by the infant when at the near-floor level. Assuming that the temporal effects of floor dust resuspension induced by the infant significantly contribute to the entirety of indoor microbial inhalation exposure in early life, an exposure mischaracterization is likely, when relying on the conventional residential dust sampling. However, the good correlation in the beta-diversity analysis-derived NMDS2 axis scores between carpet dust and IBZ samples retains the possibility that an association between carpet dust microbiota community composition and a health outcome is a proxy of microbial exposures at IBZ.

In a subsample of experiments, we also paired bulk air measurements in the ABZ with the IBZ and carpet dust determinations. We find a striking underestimation of microbial levels in the IBZ by ABZ measurements, by roughly a factor of 10 to 20, again depending on the microbial group targeted. This result is consistent with real-time particle size distribution analysis at both sampling heights in this same study, presented elsewhere [25]. In compositional terms and considering infant crawling activity, we observe a uniform separation of the bacterial composition in bulk air from the IBZ and floor dust, with a further increase of *Proteobacteria* and decrease of *Firmicutes* and *Actinobacteria* relative abundance in the bulk air compared to the IBZ. We identify a vertical gradient in the relative abundance of individual bacterial taxa: for example, *Micrococcus*, *Paracoccus*, and *Clostridium* were clearly higher in their relative abundance in the bulk air than in the IBZ air. In the absence of size-resolved microbial data and a better phylogenetic resolution to the species level, we can only speculate that small aerodynamic diameter of individual spores and limited aggregation with other spores or particles may have contributed to certain bacterial taxa staying airborne for longer periods of time. Similar to conclusions made earlier for floor dust, our study also highlights the limitations of using active air or airborne settled dust sampling as a proxy for infant inhalation exposure in the near-floor environment.

We did not observe an equally pronounced effect of a person walking over the carpets on IBZ exposure levels, when compared to crawling-induced resuspension. An earlier study found a consistent and significant effect of walking over carpeted flooring on airborne fungal levels only for carpets with high (artificial) spore loading [51]. It appears that the crawling movement generates a concentrated and localized cloud of microbe-containing particulate matter in the IBZ. This observation is matched with size-resolved total and fluorescent particulate matter monitoring in these same experiments, which are reported in separate papers [25] and (Fu et al: The Infant Playpen Effect: Crawling-Induced Dust Resuspension as a Major Source of Particulate Matter in the Infant Breathing Zone, in preparation). Enhanced air mixing induced by the adult volunteer while walking in the chamber may have majorly contributed to the smaller differences in microbial concentrations between the IBZ and ABZ compared to the crawling experiments. Also, air sampling in the IBZ during crawling sequences was done with samplers attached to the infant robot’s head, whereas the IBZ sampling was done stationary, mid carpet during walking experiments, simulating a baby located on the floor while other persons walk by. This finding is in line with previous findings about the effect of movement-induced floor dust resuspension on the exposure of small children to other indoor air pollutants [18].

Based on the results of our study, the most comprehensive assessment of infant microbial exposure to inform epidemiological studies on health effects of inhalation exposure could be obtained by combining long-term integrated active air or settled dust collection close to the IBZ with bulk air sampling in the main activity and sleeping areas of the child. There is a need for methods to easily take meaningful, repeated, or long-term integrated active air or settled dust samples from the IBZ that would take into account the effect of resuspension caused by human activities. Active air sampling typically requires battery-powered equipment with small pumps; personal active air sampling on infants would be best reflecting their exposure but, in practical terms, is not feasible. Long-term active air sampling for microbes is restricted by desiccation and degradation of microbial material over time. Recent developments in sampling approaches are promising in that respect [52, 53], but studies on the “real-life” applicability of such sampling approaches, and their representativeness of IBZ exposure, are needed.

Our experiments have some limitations with respect to simulating real-life resuspension scenarios and infant exposures. We carried out a limited set of experiments with a simplified mechanical crawling infant in a chamber to obtain samples from the IBZ and to assess the effect of crawling-induced floor dust resuspension on infant microbial exposure. This setting (a) falls short of the natural diversity and intensity of infant crawling and other near-floor activities and (b) excludes the effect of airflow patterns associated with the buoyant infant thermal plume and respiratory activities, which could affect the airborne transport of microbes around the infant. Moreover, fungal exposure scenarios were only addressed in the qPCR measurements, as active air sampling returned too many low sequence read samples in fungal ITS amplicon sequencing, which prohibited meaningful analysis of the fungal sequencing data. It should be stressed that the presented results are essentially limited by the design to the potential infant exposures by *inhalation*, while exposures by skin contact and ingestion were not addressed. We were also unable to determine size-resolved microbial concentrations as a cascade impactor was not available for the experiments. Finally, our results should be viewed in terms of microbial content associated with total suspended particulate matter (or TSP), based on the ~ 100 μm cutoff of the IOM samplers.

## Conclusions

Our results suggest that carpet dust, a commonly used surrogate sample for determinations of indoor microbial exposures, is a relatively poor proxy for the microbial content inhaled by an infant during near-floor activities. The predictive value of a carpet dust determination for IBZ exposure appears to vary between different bacterial/microbial groups. The characteristics of bacterial infant breathing zone microbiota distinct from carpet dust included lower bacterial diversity and enriched relative abundance of *Proteobacteria*, while *Firmicutes* proportion was reduced. The considerably higher microbial levels in the infant breathing zone compared to bulk room air suggest that infant crawling and subsequent resuspension of floor dust creates a concentrated and localized cloud of particulate matter around the infant which affects their associated microbial inhalation exposures. The results of this study call for advances in exposure assessment approaches that aim at understanding health effects of inhaled microbial exposure in early life.

## Additional files

Additional file 1: Table S1. QIIME mapping file. **Table S2.** Details on qPCR assays included in this study. **Table S3.** Microbial concentrations as determined with qPCR in 17 carpets used in the chamber experiments, expressed as loads (cell equivalents (CE) per square meter of carpet) and as concentrations (CE per milligram of carpet dust). **Table S4.** Means, minimum, and maximum of particulate matter (PM_100_ [μg/m^3^]) and microbial levels (cell equivalents/m^3^) in infant breathing zone (IBZ) during five individual crawling and walking experiments on different rugs and corresponding ratios of crawling versus walking resuspension experiments. **Table S5.** Spearman rank-order correlations between the top 10 most abundant bacterial genera in infant breathing zone air and carpet dust. **Table S6.** Differential features (OTUs) in carpet dust versus infant breathing zone air samples as determined via LEfSe analysis of 16 carpet dust and 16 corresponding IBZ samples. **Table S7.** Dust load, quantitative PCR measurements, and particle mass concentrations from crawling experiments on 17 carpets. **Table S8.** Dust load, quantitative PCR measurements, and particle mass concentrations from crawling and walking experiments on 5 carpets. (XLSX 72 kb)

Additional file 2: Concentrations of bacterial and fungal groups determined with qPCR in 17 carpets (expressed as cell equivalents (CE) per square meter of carpet) (A) and corresponding infant breathing zone levels (CE per cubic meter of air) (B) during crawling sequences. (TIFF 62 kb)

Additional file 3: Spearman rank-order correlations between dust load (g/m^2^ of carpet) and microbial concentrations (CE/mg) in carpet dust versus particulate matter (μg/m^3^ of sampled air) and microbial concentrations (CE/m^3^ of sampled air) in the infant breathing zone during crawling experiments on 17 carpets. Microbial concentrations were determined with qPCR. (PNG 58 kb)
