# Supplementary figures and images for: Crawling-induced floor dust resuspension affects the microbiota of the infant breathing zone
Source: Microbiome. 2018 Feb 2;6:25. doi: 10.1186/s40168-018-0405-8 (PMC5797336; doi:10.1186/s40168-018-0405-8)

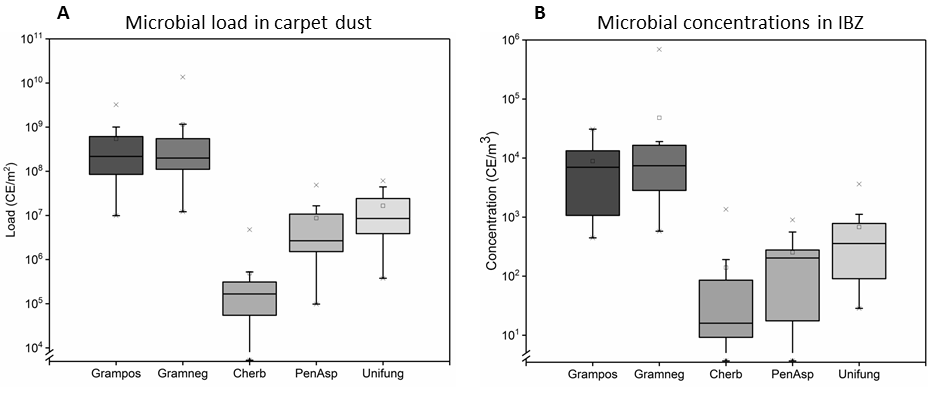

Supplement: Supplementary file 2 — Concentrations of bacterial and fungal groups determined with qPCR in 17 carpets (expressed as cell equivalents (CE) per square meter of carpet) (A) and corresponding infant breathing zone levels (CE per cubic meter of air) (B) during crawling sequences. (TIFF 62 kb) [file 40168_2018_405_MOESM2_ESM.tif]

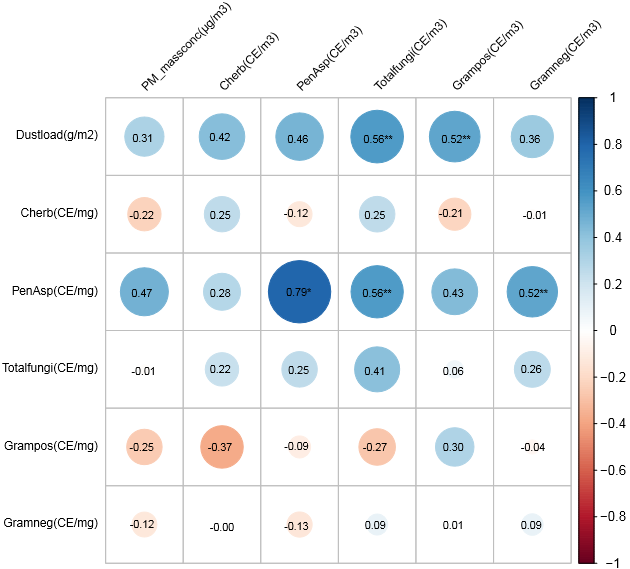

Supplement: Supplementary file 3 — Spearman rank-order correlations between dust load (g/m2 of carpet) and microbial concentrations (CE/mg) in carpet dust versus particulate matter (μg/m3 of sampled air) and microbial concentrations (CE/m3 of sampled air) in the infant breathing zone during crawling experiments on 17 carpets. Microbial concentrations were determined with qPCR. (PNG 58 kb) [file 40168_2018_405_MOESM3_ESM.png]
